# Cardiovascular Disease Patterns, Mortality, and Hospitalization Trends in Adults Over 18: Insights From the Behavioral Risk Factor Surveillance System Database

**DOI:** 10.7759/cureus.66604

**Published:** 2024-08-10

**Authors:** Okelue E Okobi, Enyioma Nwogwugwu, Cosmas O Ihezie, Olutayo O Olasupo, Christopher I Emovon, Hassana Wambai-Sani, Oboatarhe B Ezie, Akinbanji R Afolabi, Okechukwu C Erinne, Rachel A O’dare

**Affiliations:** 1 Family Medicine, Larkin Community Hospital Palm Springs Campus, Miami, USA; 2 Family Medicine, Medficient Health Systems, Laurel, USA; 3 Family Medicine, Lakeside Medical Center, Belle Glade, USA; 4 Internal Medicine, Lincoln Medical and Mental Health Center, New York, USA; 5 Orthopedics, Federal University Teaching Hospital, Owerri, Owerri, NGA; 6 Internal Medicine, Phoenix Rehabilitation and Nursing Center, New York, USA; 7 Internal Medicine, Wyckoff Heights Medical Center, New York, USA; 8 Family Medicine, Windsor University School of Medicine, Basseterre, KNA; 9 Family Medicine, University of Benin, Benin City, NGA; 10 Medicine, Emory University, Atlanta, USA; 11 Endocrinology, Children's Hospital of Philadelphia, Philadelphia, USA; 12 Epidemiology, University of Texas Health Science Center at Houston, Houston, USA; 13 Nursing, South University, Savannah, Savannah, USA; 14 General Medicine, Medical University of Graz, Graz, AUT

**Keywords:** brfss database, adults, trends, chronic health indicators, hospitalization, mortality, cvd medicine pattern

## Abstract

Background

Cardiovascular diseases (CVDs), including coronary artery disease, heart attacks, strokes, and hypertension, are the leading cause of global morbidity and mortality. Despite advancements in diagnostic techniques, treatment protocols, and public health initiatives, the prevalence of CVD continues to rise. Hence, understanding trends of predisposing factors for CVD and current treatment modalities such as medication use and frequency of hospitalization is essential for developing effective interventions and improving public health strategies. This study leverages Behavioral Risk Factor Surveillance System (BRFSS) data to analyze these trends among adults older than 18 years.

Methods

Data were sourced from the BRFSS database, analyzing CVD patterns from 2019 to 2021. The study included adults with high cholesterol or blood pressure, coronary heart disease, stroke, and heart failure. Data analysis utilized age-adjusted prevalence, mortality, and hospitalization rates.

Results

The analysis of the BRFSS data revealed several key trends in CVD patterns from 2019 to 2021. There was a statistically significant increase (p<0.05) in the age-adjusted prevalence of adults taking medication for high cholesterol, rising from 28.9% to 31%, and for controlling high blood pressure, increasing from 57.7% to 60.4%. From 2019 to 2021, coronary heart disease mortality increased from 360,900 to 382,820, while stroke deaths rose from 150,005 to 162,890. Trends show rising mortality for both conditions despite missing data for some years. Mortality rates for coronary heart disease and stroke also rose and were statistically significant (p<0.05), with coronary heart disease mortality increasing from 88 to 92.8 cases per 100,000, and stroke mortality from 37 to 41.1 cases per 100,000. Hospitalization rates for heart failure among Medicare beneficiaries aged 65 and older initially decreased in 2020, likely due to the COVID-19 pandemic impacting hospital admissions, but rose again in 2021 as healthcare-seeking behaviors normalized. Significant gender and racial disparities were observed, with higher mortality rates among males (127.4 per 100,000) and Black, non-Hispanic individuals (110.5 per 100,000).

Conclusions

This study highlights the increasing medication use for cholesterol and blood pressure among adults older than 18 years, yet mortality rates for coronary heart disease and stroke persist. Significant gender and racial disparities in medication use and mortality rates were observed. These findings underscore the need for targeted public health intervention towards improving medication adherence and addressing social determinants of health, to reduce CVD burden and enhance health equity across diverse populations.

## Introduction

Cardiovascular diseases (CVDs) encompass a broad range of conditions affecting the heart and blood vessels, including coronary artery disease, heart attacks, strokes, heart failure, arrhythmias, and hypertension [1]. CVDs are the leading cause of death worldwide, claiming an estimated 17.9 million lives in 2019, with 85% of these deaths due to heart attacks and strokes [1-3]. Prevention of CVDs largely involves addressing behavioral and environmental risk factors, and early detection is crucial for effective management. Despite medical advancements, the prevalence and impact of CVDs continue to rise, particularly among adults, making CVD a significant public health challenge. Understanding the patterns of CVD medication use, mortality, and hospitalization trends is essential for developing effective strategies to address this health crisis [4,5].

CVDs often share common risk factors, including hypertension, diabetes, obesity, smoking, and physical inactivity [1,4]. In addition, the management of CVD is multifaceted comprising lifestyle modification, pharmacotherapy, and in some cases, surgical interventions [6,7]. The effective use of cardiovascular medications, such as antihypertensives, antiplatelets, and statins, is pivotal in controlling disease progression, preventing and reducing adverse outcomes [6-8].

Analyzing medication patterns provides valuable insights into adherence to clinical guidelines and the effectiveness of public health interventions aimed at promoting cardiovascular health. It also sheds light on potential gaps in treatment and disparities in access to healthcare services. Furthermore, examining mortality and hospitalization trends offers a comprehensive picture of the burden of CVD on individuals and healthcare systems. Mortality data highlight the ultimate impact of these diseases, while hospitalization rates reflect the frequency and severity of acute cardiovascular events requiring medical attention [9,10].

In recent years, there has been a growing emphasis on preventive cardiology and implementing public health policies to reduce the burden of CVDs. These include initiatives to promote healthy lifestyles, enhance access to preventive services, and improve the management of chronic conditions. The insights gained from this study will contribute to the evidence base supporting these initiatives and inform the development of targeted interventions [11,12].

The Behavioral Risk Factor Surveillance System (BRFSS) employs rigorous quality assurance measures, including regular data audits, interviewer training programs, and validation studies that compare BRFSS data with other national surveys to maintain high data accuracy and reliability. This rich source of information enables researchers to analyze various aspects of public health, including the epidemiology of CVDs [13-15]. By examining these aspects, the study aims to uncover the effectiveness of current pharmacotherapy in managing CVDs, identify demographic and socioeconomic disparities in treatment and outcomes, and provide insights into the prevalence and impact of cardiovascular conditions on public health.

## Materials and methods

Data source and study design

This study aims to analyze patterns of CVD medication usage, mortality, and hospitalization trends among adults, utilizing data from the BRFSS database, a comprehensive, ongoing health survey managed by the Centers for Disease Control and Prevention (CDC). The BRFSS collects data on health-related risk behaviors, chronic health conditions, and the use of preventive services. Established in 1984, the BRFSS is the world's largest continuous health survey system, operating nationwide across all states, the District of Columbia, and three territories. Over 400,000 annual adult interviews provide a comprehensive overview of health-related behaviors and conditions [14]. Our study focused on data collected between 2019 and 2021, leveraging its cross-sectional design to analyze CVD medication usage trends, mortality, and hospitalization rates.

Study participants and inclusion criteria

The study population included adults aged 18 years and older who participated in the BRFSS surveys conducted in 2019 and 2021. The inclusion criteria for our study specifically required participants to have complete data on CVD-related questions, including medication usage for high cholesterol and high blood pressure, as well as information on coronary heart disease and stroke mortality. Additionally, data from Medicare beneficiaries aged 65 years and older were included to analyze hospitalization rates for heart failure. The study exclusively excluded participants with incomplete data or records with inconsistencies or errors in the reported data that cannot be resolved through data validation processes.

Data collection, quality assurance and analysis, and statistical methods

Data collection for the BRFSS involves random-digit dialing of landline and cellular telephones to gather health-related information from non-institutionalized U.S. adults. Standardized questionnaires are administered by trained interviewers to ensure consistency and reliability. The BRFSS employs rigorous quality assurance measures, including regular data audits, interviewer training programs, and validation studies, to maintain high data accuracy and reliability.

Data analysis was performed using the Statistical Package for the Social Sciences (IBM SPSS Statistics for Windows, IBM Corp., Version 29.0, Armonk, NY). Descriptive statistics summarized demographic characteristics and prevalence rates. Age-adjusted prevalence rates, annual percent change, and 95% confidence intervals (CIs) were calculated for medication usage and mortality rates. Trends over the three years were assessed using linear regression models. Subgroup analyses by gender and race/ethnicity were conducted to identify disparities, as these factors are known to significantly influence CVD outcomes and access to healthcare. Heart failure hospitalization rates were reported per 1,000 Medicare beneficiaries, with weighted proportions and 95% CIs computed utilizing SPSS statistical software packages. Chi-square tests were conducted to assess the significance of trends and differences in medication usage and mortality rates. With a p-value greater than 0.05, the results were considered not statistically significant.

Ethical considerations

The BRFSS data are publicly available and de-identified, ensuring participant confidentiality and compliance with ethical standards. The study adhered to ethical secondary data analysis guidelines, ensuring no individual participants could be identified. As the BRFSS is conducted by the CDC, it operates under federal regulations to protect human subjects, including Institutional Review Board (IRB) approvals and informed consent processes. No additional IRB approval was required for this secondary analysis.

## Results

Taking medicine for high cholesterol among adults (>18 years)

Analyzing CVD patterns using BRFSS data reveals trends in medication use, mortality, and demographic disparities. This section details findings on medication for high cholesterol and blood pressure and mortality rates for coronary heart disease and stroke.

The prevalence of adults taking medication for high cholesterol showed a significant increase from 2019 to 2021. In 2019, 28.9% of adults reported taking medicine for high cholesterol, with a 95% CI of 28.6% to 29.2%. By 2021, this prevalence had increased to 31% (95% CI: 30.6% to 31.3%). The annual percent change between these two datasets is 7.2% (95% CI: 7.1-7.3%). This overall increase suggests improvements in detecting and managing high cholesterol levels, which is crucial for reducing the risk of cardiovascular events.

Gender Differences

Gender-specific data reveal that males had a higher prevalence of cholesterol medication usage compared to females. In 2019, 30.5% of males (95% CI: 30.0% to 30.9%) reported taking cholesterol medication, compared to 27.4% of females (95% CI: 27.0% to 27.8%). By 2021, these figures rose to 32.4% (95% CI: 31.9% to 32.8%) for males and 29.6% (95% CI: 29.2% to 30.1%) for females. For males, the annual percent change between these two datasets is 6.2% (95% CI: 5.9-6.3%) as compared to 8.0% (95% CI: 7.9-8.3%) in females. This increase in both genders indicates a positive trend towards better cholesterol management but also highlights the need for targeted interventions to address the slightly lower uptake among females.

Racial/Ethnic Differences

The data also highlight significant differences in medication usage across racial and ethnic groups. In 2021, Hispanic adults had the highest prevalence of taking medication for high cholesterol at 32.2% (95% CI: 31.2% to 33.2%), followed by White, non-Hispanic adults at 30.8% (95% CI: 30.4% to 31.2%). Black, non-Hispanic adults reported a prevalence of 29.2% (95% CI: 28.3% to 30.1%), while Asian, non-Hispanic adults had a prevalence of 31.6% (95% CI: 29.4% to 33.9%). These variations suggest the need for culturally tailored interventions to improve medication adherence and cholesterol management among different racial and ethnic groups.

Medication adherence for hypertension management among adults (>18 years)

The use of medication to control high blood pressure among adults with diagnosed hypertension also showed an upward trend from 2019 to 2021. In 2019, 57.7% of adults with high blood pressure took medication to control their condition (95% CI: 57.1% to 58.4%). This increased to 60.4% in 2021 (95% CI: 59.6% to 61.1%). The annual percent change between these two datasets is 4.7% (95% CI: 4.6-4.9%). This positive trend indicates an improvement in the management of hypertension, which is a major risk factor for both coronary heart disease and stroke (Table 1).

**Table 1:** Cardiovascular disease medication use pattern - indicates data not available.

Indicators	Taking medicine for high cholesterol among adults				Taking medicine to control high blood pressure among adults with high blood pressure			
Characteristics	2019		2021		2019		2021	
Age-adjusted prevalence (%)	%	95% CI	%	95% CI	%	95% CI	%	95% CI
Overall	28.9	28.6-29.2	31	30.6-31.3	57.7	57.1-58.4	60.4	59.6-61.1
Male	30.5	30.00-30.9	32.4	31.9-32.8	55	54.2-55.8	57.3	56.4-58.1
Female	27.4	27.00-27.8	29.6	29.2-30.1	61.8	60.7-62.9	65.2	63.9-66.4
White, non-Hispanic	28.9	28.5-29.2	30.8	30.4-31.2	56.1	55.4-56.8	59.6	58.8-60.4
Black, non-Hispanic	27.8	26.9-28.7	29.2	28.3-30.1	64.3	62.4-66.1	67.5	65.4-69.5
Hispanic	29.3	28.4-30.3	32.2	31.2-33.2	56.8	55.00-58.6	56.4	54.6-58.3
Asian, non-Hispanic	-	-	31.6	29.4-33.9	-	-	-	-
Hawaiian or Pacific Islander, non-Hispanic	-	-	-	-	-	-	-	-
American Indian or Alaska Native, non-Hispanic	-	-	-	-	-	-	-	-
Multiracial, non-Hispanic	29.9	27.9-32.00	32.2	30.00-34.5	-	-	-	-

The first group focuses on adults with high cholesterol who are managing their condition by taking prescribed medication. This population includes those who have been diagnosed with high cholesterol after testing and are actively using medication to control it. The data stratifies individuals based on their engagement in treatment. The second group targets adults with high blood pressure who are taking medication to manage their condition. This group consists of those diagnosed with hypertension and taking prescribed medicine. The primary distinction between these groups lies in the specific condition being managed-cholesterol versus blood pressure-and their adherence to medical advice.

Gender Differences

In 2019, 55% of males with high blood pressure (95% CI: 54.2% to 55.8%) reported taking medication to control it, compared to 61.8% of females (95% CI: 60.7% to 62.9%). By 2021, these figures had increased to 57.3% (95% CI: 56.4% to 58.1%) for males and 65.2% (95% CI: 63.9% to 66.4%) for females. For males, the annual percent change between these two datasets is 4.2% (95% CI: 4.0-4.3%) as compared to 5.6% (95% CI: 5.2-5.9%) in females. This data shows that females are more likely than males to take medication for high blood pressure, highlighting the need for strategies to improve medication adherence among males.

Racial/Ethnic Differences

Significant racial and ethnic disparities were observed in using medication to control high blood pressure. In 2021, Black, non-Hispanic adults had the highest prevalence at 67.5% (95% CI: 65.4% to 69.5%), indicating relatively better control among this group. Hispanic adults had a prevalence of 56.4% (95% CI: 54.6% to 58.3%), while White, non-Hispanic adults reported a prevalence of 59.6% (95% CI: 58.8% to 60.4%). Asian, non-Hispanic adults had the lowest prevalence at 31.6% (95% CI: 29.4% to 33.9%). These disparities underscore the importance of addressing the social determinants of health and ensuring equitable access to healthcare and medication for all racial and ethnic groups.

Mortality trends

Coronary Heart Disease Mortality Trends

The age-adjusted mortality rate for coronary heart disease exhibited an upward trajectory from 2019 through 2021, underscoring persistent challenges despite advancements in medication usage. In 2019, the mortality rate was 88 cases per 100,000 population (95% CI: 87.7 to 88.3). By 2020, it increased to 91.8 cases per 100,000 (95% CI: 91.5 to 92.1) and further rose to 92.8 cases per 100,000 in 2021 (95% CI: 92.5 to 93.1). This trend highlights coronary heart disease's ongoing public health burden and emphasizes the critical need for enhanced prevention, early detection, and treatment strategies (Table 2).

**Table 2:** Cardiovascular disease mortality trends - indicates data not available.

Indicator	Coronary heart disease mortality among all people, the underlying cause						Cerebrovascular disease (stroke) mortality among all people, the underlying cause					
Year	2019		2020		2021		2019	2020	2021	2019	2020	2021
Overall (number)	360900	-	382820	-	375476	-	150005	-	160264	-	162890	-
Cases per 100,000	Cases per 100,000	95% CI	Cases per 100,000	95% CI	Cases per 100,000	95% CI	Cases per 100,000	95% CI	Cases per 100,000	95% CI	Cases per 100,000	95% CI
Overall	88	87.7-88.3	91.8	91.5-92.1	92.8	92.5-93.1	37	36.8-37.1	38.8	38.6-39.00	41.1	40.9-41.3
Male	120.9	120.3-121.4	126.2	125.7-126.7	127.4	126.8-127.9	37.6	37.3-37.9	39.8	39.5-40.1	41.5	41.2-41.8
Female	61.7	61.4-62.00	64.1	63.7-64.4	64.7	64.4-65.1	35.8	35.6-36.1	37.4	37.2-37.7	40.2	39.9-40.5
White, non-Hispanic	91	90.7-91.3	93.4	93.00-93.7	96.2	95.8-96.6	35.7	35.5-35.9	37.2	37.00-37.4	39.8	39.6-40.1
Black, non-Hispanic	103.9	102.8-104.9	115.8	114.7-116.9	110.5	109.5-111.6	53.1	52.4-53.9	57.6	56.8-58.3	59.6	58.8-60.4
Hispanic	67.8	66.9-68.6	75.6	74.7-76.5	70.6	69.7-71.5	32.8	32.2-33.4	34.9	34.3-35.5	36.1	35.4-36.7
Asian, non-Hispanic	49.1	48.1-50.1	54.8	53.8-55.9	52.7	51.7-53.7	29.3	28.5-30.1	31.6	30.8-32.3	32.6	31.8-33.4
Hawaiian or Pacific Islander, non-Hispanic	97.4	88.6-106.2	97	88.4-105.6	98.8	90.2-107.4	50.9	44.3-57.6	47	40.9-53.2	46.7	40.7-52.8
American Indian or Alaska Native, non-Hispanic	79.4	75.6-83.2	82.6	78.9-86.4	81.7	78.00-85.4	31.4	29.00-33.8	34	31.5-36.4	34.6	32.1-37.1
Multiracial, non-Hispanic	38.3	36.2-40.5	42.1	39.9-44.3	40.7	38.5-42.8	17.8	16.3-19.3	18.9	17.4-20.4	20.3	18.8-21.8

Gender disparities: Gender-specific data underscore significant disparities in coronary heart disease mortality rates. In 2021, males exhibited a notably higher mortality rate of 127.4 per 100,000 population (95% CI: 126.8 to 127.9) compared to females, whose mortality rate was 64.7 per 100,000 (95% CI: 64.4 to 65.1). This stark contrast signals the necessity for targeted interventions aimed at reducing mortality among males, potentially linked to higher exposure to risk factors or disparities in healthcare utilization.

Racial/ethnic disparities: Racial and ethnic disparities in coronary heart disease mortality rates are also evident. In 2021, Black, non-Hispanic individuals recorded the highest mortality rate at 110.5 cases per 100,000 (95% CI: 109.5 to 111.6), followed by White, non-Hispanic individuals at 96.2 cases per 100,000 (95% CI: 95.8 to 96.6). Hispanic individuals reported a mortality rate of 70.6 per 100,000 (95% CI: 69.7 to 71.5), whereas Asian, non-Hispanic individuals had the lowest rate at 52.7 per 100,000 (95% CI: 51.7 to 53.7). These disparities necessitate tailored public health strategies to address specific risk factors and improve outcomes across diverse racial and ethnic groups.

Cerebrovascular Disease (Stroke) Mortality

The age-adjusted mortality rate for CVD (stroke) also showed an increasing trend from 2019 to 2021. In 2019, the stroke mortality rate was 37 cases per 100,000 population (95% CI: 36.8 to 37.1). This increased to 38.8 cases per 100,000 in 2020 (95% CI: 38.6 to 39.0) and further to 41.1 cases per 100,000 in 2021 (95% CI: 40.9 to 41.3). This trend underscores the ongoing public health burden of stroke and the importance of improving preventive measures and acute care (Table 2).

Gender differences: The mortality rate for males was slightly higher than for females. In 2021, the rate for males was 41.5 cases per 100,000 (95% CI: 41.2 to 41.8), compared to 40.2 cases per 100,000 for females (95% CI: 39.9 to 40.5). This small but significant difference suggests the need for focused stroke prevention efforts among males.

Racial/ethnic differences: Disparities in stroke mortality rates were also observed among different racial and ethnic groups. Black, non-Hispanic adults had the highest stroke mortality rate in 2021 at 59.6 cases per 100,000 population (95% CI: 58.8 to 60.4), indicating a significant burden of cerebrovascular disease in this group. Hispanic adults had a stroke mortality rate of 36.1 cases per 100,000 (95% CI: 35.4 to 36.7), while White, non-Hispanic adults had a rate of 39.8 cases per 100,000 (95% CI: 39.6 to 40.1). Asian, non-Hispanic adults had the lowest stroke mortality rate at 32.6 cases per 100,000 (95% CI: 31.8 to 33.4). These disparities point to the necessity of targeted stroke prevention and treatment programs tailored to the unique risk profiles of different racial and ethnic groups.

Hospitalization trends for heart failure among Medicare beneficiaries

From 2019 to 2021, hospitalizations for heart failure as the principal diagnosis among Medicare beneficiaries aged 65 and older showed dynamic trends. Initially, hospitalizations declined significantly from 805,708 in 2019 to 661,335 in 2020, followed by an increase to 725,404 in 2021. This pattern was mirrored in cases per 1,000 beneficiaries, decreasing from 27.72 (95% CI: 27.66-27.78) in 2019 to 22.87 (95% CI: 22.81-22.92) in 2020, then rising to 25.91 (95% CI: 25.85-25.97) in 2021 (Table 3).

**Table 3:** Heart failure among Medicare beneficiaries hospitalization trends - indicates data not available.

Indicator	Hospitalization trends for heart failure among Medicare beneficiaries					
Year	2019		2020		2021	
Overall (number)	805708	-	661335	-	725404	-
cases per 100,000	cases per 1,000	95% CI	cases per 1,000	95% CI	cases per 1,000	95% CI
Overall	27.72	27.66-27.78	22.87	22.81-22.92	25.91	25.85-25.97
Male	29.92	29.83-30.02	24.57	24.48-24.65	27.39	27.3-27.48
Female	25.95	25.88-26.03	21.47	21.4-21.54	24.67	24.59-24.75
White, non-Hispanic	25.62	25.56-25.68	21.05	20.99-21.1	23.83	23.77-23.89
Black, non-Hispanic	55.46	55.15-55.76	48.18	47.88-48.47	55.85	55.52-56.18
Hispanic	36.31	35.79-36.84	29.91	29.43-30.4	33.68	33.16-34.2
American Indian or Alaska Native, non-Hispanic	26.46	25.62-27.3	23.93	23.13-24.74	24.28	23.45-25.12
Asian or Pacific Islander, non-Hispanic	20.04	19.7-20.39	15.54	15.24-15.85	18.4	18.06-18.73

Gender Differences

Examining gender disparities, males consistently had higher hospitalization rates than females. In 2019, males had 29.92 (95% CI: 29.83-30.02) cases per 1,000, decreasing to 24.57 (95% CI: 24.48-24.65) in 2020 and rising to 27.39 (95% CI: 27.30-27.48) in 2021. Females started at 25.95 (95% CI: 25.88-26.03) in 2019, dropped to 21.47 (95% CI: 21.40-21.54) in 2020, and increased to 24.67 (95% CI: 24.59-24.75) in 2021.

Racial/Ethnic Differences

Racial and ethnic disparities were stark, with White, non-Hispanic individuals having the lowest rates: 25.62 (95% CI: 25.56-25.68) in 2019, 21.05 (95% CI: 20.99-21.10) in 2020 and 23.83 (95% CI: 23.77-23.89) in 2021. Black, non-Hispanic individuals had the highest rates, starting at 55.46 (95% CI: 55.15-55.76) in 2019, decreasing to 48.18 (95% CI: 47.88-48.47) in 2020, and increasing slightly to 55.85 (95% CI: 55.52-56.18) in 2021. Hispanic individuals showed intermediate rates: 36.31 (95% CI: 35.79-36.84) in 2019, 29.91 (95% CI: 29.43-30.40) in 2020, and 33.68 (95% CI: 33.16-34.20) in 2021. Asian or Pacific Islander, non-Hispanic individuals had the lowest rates: 20.04 (95% CI: 19.70-20.39) in 2019, 15.54 (95% CI: 15.24-15.85) in 2020, and 18.40 (95% CI: 18.06-18.73) in 2021.

These findings highlight fluctuating trends in heart failure hospitalization rates among older Medicare beneficiaries, revealing significant disparities based on gender and race/ethnicity. Targeted interventions are crucial to mitigate these disparities and enhance cardiovascular health outcomes across all demographic groups.

## Discussion

The analysis of CVD patterns based on data from the BRFSS identifies notable trends and disparities in medication usage and mortality rates, offering critical insights for public health interventions and policies aimed at reducing the CVD burden. CVDs remain the top global killer, with rising prevalence and mortality rates. A study by Borkowski et al. highlights that non-Hispanic Black and Hispanic populations face higher CVD risks, exacerbated by socioeconomic barriers such as lower income, education, and insurance coverage. Addressing these factors through tailored interventions and comprehensive socioeconomic data is crucial for improving cardiovascular health outcomes [16].

Comparing the medication usage trends between 2019 and 2021 with historical data from 2003 to 2012, the recent increase in adults taking medication for high cholesterol (from 28.9% to 31%) and high blood pressure (from 57.7% to 60.4%) reflects a continuing trend observed over the previous decade [17]. During 2003-2012, the percentage of adults aged 40 and over using prescription cholesterol-lowering medications rose from 20% to 25%. This increase in cholesterol-lowering medication use aligns with the general rise in medication usage over time. Additionally, in 2011-2012, a majority of those using cholesterol-lowering medications reported using statins, reflecting the ongoing emphasis on statin therapy in managing high cholesterol [17]. This uptick suggests enhanced awareness, diagnosis, and management of these conditions, crucial for preventing cardiovascular events [18].

These findings are consistent with previous studies which reported that gender differences persist in medication usage, with females more likely than males to take medication for high blood pressure, while males show higher rates for high cholesterol medication [19]. These patterns underscore the necessity for gender-specific strategies, such as tailored patient education programs and targeted adherence support, to bolster medication adherence and health outcomes [20]. Racial and ethnic disparities in medication usage highlight the need for tailored interventions. Hispanic adults exhibited the greatest increase in medication usage for high cholesterol, whereas Black, non-Hispanic adults had the highest prevalence of high blood pressure medication usage. The comparative data from 1999 to 2020 reveals notable racial and ethnic disparities in medication usage. Hispanic and Latino adults experienced the most significant increase in statin use, with a rise of 17.12 percentage points, reflecting their substantial growth in high cholesterol medication usage. In contrast, Black, non-Hispanic adults showed the highest prevalence of angiotensin-converting enzyme inhibitors (ACEI) and angiotensin receptor blockers (ARB), with significant utilization but no marked increase in high blood pressure medication usage compared to other groups. Despite improvements, substantial disparities in medication usage and optimal care persist across racial and ethnic groups [21,22]. Despite these efforts, Black, non-Hispanic adults also had the highest mortality rates for coronary heart disease and stroke, underscoring the multifaceted nature of health disparities that medication alone cannot fully address. Addressing broader social determinants of health, such as access to healthcare and socioeconomic status, is imperative for improving outcomes in these populations [19,21].

This study's findings reported that mortality rates for coronary heart disease and stroke increased despite improvements in medication usage. Coronary heart disease mortality rose from 88 to 92.8 cases per 100,000 population, and stroke mortality increased from 37 to 41.1 cases per 100,000 population. These trends emphasize the ongoing challenge of managing cardiovascular health beyond pharmacological interventions, necessitating comprehensive approaches to address lifestyle factors and ensure equitable access to quality care [23,24].

Gender disparities in mortality rates persist, with males consistently experiencing higher rates for both coronary heart disease and stroke compared to females. This underscores the urgency of targeted interventions aimed at reducing risk factors such as smoking and promoting healthier lifestyles among males [23-25]. Racial and ethnic disparities in mortality rates remain significant, with Black, non-Hispanic adults bearing the highest burden for both coronary heart disease and stroke despite relatively high rates of high blood pressure medication usage. These disparities highlight the critical role of social determinants of health, including healthcare access and quality of care, in shaping health outcomes. Tailored interventions, such as community-based health programs and improved access to culturally competent healthcare, are essential to mitigate mortality disparities among minority populations [26,27].

Analysis of hospitalization rates for heart failure among Medicare beneficiaries aged 65 years and older from 2019 to 2021 reveals significant fluctuations influenced by external factors, particularly the COVID-19 pandemic. In 2019, the hospitalization rates were relatively stable, reflecting pre-pandemic patterns of healthcare utilization. However, in 2020, there was a notable decline in hospitalizations, a trend that aligns with widespread disruptions in healthcare services due to the pandemic. During this period, many healthcare facilities faced unprecedented challenges, and patients may have been reluctant to seek medical care due to fears of COVID-19 exposure or restrictions imposed on elective procedures and non-COVID-related hospital admissions. As the pandemic continued, shifts in healthcare utilization patterns became apparent, with a reduction in routine and elective hospital admissions. This decline was attributed to both patient avoidance of hospitals and changes in the availability of medical services. For instance, many hospitals were repurposed to handle COVID-19 cases, impacting the ability to treat non-COVID conditions like heart failure. Additionally, public health guidelines and lockdown measures may have contributed to reduced hospital visits [28].

Analysis of hospitalization rates for heart failure among Medicare beneficiaries aged 65 years and older from 2019 to 2021 reveals fluctuating trends influenced by external factors such as the COVID-19 pandemic. Hospitalizations declined notably in 2020, coinciding with pandemic-related changes in healthcare utilization patterns, followed by an increase in 2021 as healthcare-seeking behaviors normalized post-pandemic [28].

Gender disparities persist in hospitalization rates, with males consistently experiencing higher rates compared to females, reflecting established higher heart failure risks among men [29]. These findings underscore the need for targeted preventive strategies and improved access to care for male populations. Racial and ethnic disparities are evident, with Black, non-Hispanic individuals exhibiting significantly higher hospitalization rates for heart failure compared to White, non-Hispanic, and Asian or Pacific Islander individuals. These disparities highlight broader inequities influenced by socioeconomic factors and structural racism, necessitating culturally sensitive healthcare approaches for effective mitigation [30]. Conversely, Asian or Pacific Islander, non-Hispanic individuals demonstrate lower hospitalization rates, suggesting potential protective factors that merit further investigation to sustain optimal heart health outcomes.

Strengths and limitations

The study leverages the BRFSS database, one of the largest ongoing health surveys globally, ensuring a robust and diverse sample size that enhances the generalizability of findings across various demographic groups. The BRFSS's comprehensive data collection on health-related risk behaviors, chronic conditions, and preventive service utilization offers a detailed perspective on CVD patterns and trends. The detailed demographic breakdown by gender and race/ethnicity highlights specific disparities, underscoring the need for tailored public health interventions and providing actionable data for policymakers.

However, the study has several limitations. Reliance on self-reported data introduces potential biases, and the cross-sectional nature of the data limits the ability to establish causality. The absence of detailed clinical data, such as specific cholesterol or blood pressure levels, restricts understanding of disease severity and management effectiveness. Acknowledging these limitations, future research can build upon these findings to refine strategies for combating CVD and achieving health equity.

## Conclusions

In conclusion, the BRFSS data analysis reveals significant increases in medication usage for managing high cholesterol and high blood pressure among adults, alongside rising mortality rates for coronary heart disease and stroke. Gender and racial/ethnic disparities, particularly affecting Black, non-Hispanic individuals despite higher medication usage, underscore persistent challenges in public health. These findings, including the rising prevalence of CVD despite increased medication usage and significant gender and racial disparities in outcomes, emphasize the urgency of targeted interventions and policies that improve medication adherence, address social determinants of health, promote healthy behaviors, and enhance early detection and management. Addressing these issues is essential for improving medication adherence, tackling social determinants of health, promoting healthy behaviors, and enhancing early detection and management. Recent healthcare initiatives are increasingly focused on these areas to reduce disparities and improve overall cardiovascular health outcomes. Tailoring interventions to specific populations is crucial for reducing the burden of CVD and achieving equitable healthcare access, thereby fostering better health outcomes across diverse communities.
